# A study of standardized extracts of *Picrorhiza kurroa* Royle ex Benth in experimental nonalcoholic fatty liver disease

**DOI:** 10.4103/0975-9476.72622

**Published:** 2010

**Authors:** Sapna N. Shetty, Sushma Mengi, Rama Vaidya, Ashok D. B. Vaidya

**Affiliations:** *Medical Research Centre, Kasturba Health Society, Mumbai, India*

**Keywords:** Hepatoprotective, high fat diet, insulin resistance, metabolic syndrome, non-alcoholic fatty liver, *Picrorhiza kurroa* reverse pharmacology

## Abstract

As a major organ of intermediary metabolism, the liver is exposed to a variety of metabolic insults due to diseases and xenobiotics viz., insulin resistance (IR) drugs, toxins, microbial products, etc. One of the consequences of these metabolic insults including obesity and type 2 diabetes mellitus is the development of non-alcoholic fatty liver disease (NAFLD). The recent alarming increase in the prevalence of NAFLD compels the need to develop an appropriate animal model of the disease so as to evolve effective interventions. In this study, we have developed, in the rat, a new model of NAFLD showing several key features akin to the disease in humans. Male Wistar rats were challenged with 30% high fat diet (HFD) – butter, for 2 weeks to induce NAFLD. A hydroalcoholic extract of *Picrorhiza kurroa* was administered to study the possible reversal of fatty changes in the liver. *The extract* was given in two doses viz., 200mg/kg and 400 mg/kg b.i.d., p.o. for a period of 4 weeks. There were three control groups (n = 6/group) – vehicle with a regular diet, vehicle with HFD, and HFD with silymarin – a known hepatoprotective.

Histopathology showed that the *P. kurroa* extract brought about a reversal of the fatty infiltration of the liver (mg/g) and a lowering of the quantity of hepatic lipids (mg/g) compared to that in the HFD control group (38.33 ± 5.35 for 200mg/kg; 29.44 ± 8.49 for 400mg/kg of *P. kurroa* vs.130.07 ± 6.36mg/g of liver tissue in the HFD control group; *P*<0.001). Compared to the standard dose of the known hepatoprotective silymarin, *P. kurroa* reduced the lipid content (mg/g) of the liver more significantly at the dose of 400mg/kg (57.71 ± 12.45mg/kg vs. 29.44 ± 8.49 for the silymarin group vs. 400mg/kg of *P. kurroa*, *P*<0.001). In view of the increasing prevalence of metabolic syndrome and NAFLD, *P. kurroa* should be investigated by the reverse pharmacology path as a potential drug for the treatment of NAFLD, and essential safety studies and preformulation research for concentration of the putative actives should be carried out.

## INTRODUCTION

Nonalcoholic fatty liver disease (NAFLD) has now emerged as a global health challenge. In 1980, Ludwig *et al*, noticed fatty infiltration in the liver biopsy of nonalcoholic patients.[1] Later, NAFLD was observed in diabetes mellitus (DM) and obesity.[2,3] In type 2 diabetes, NAFLD occurs in up to 75% of patients.[4] In obesity, NAFLD and hepatic fibrosis are prevalent.[5] In fact, DM is an independent risk factor for liver-related deaths in NAFLD.[6] Obesity and DM are often accompanied by insulin dysregulation of glucose and lipid metabolism. Potentially hepatotoxic fatty acids in hyperinsulinemic states can lead to NAFLD. Obesity per se is a risk factor of NAFLD: visceral fat is a predictor of hepatic steatosis.[7] Reduction in visceral fat mass has also been shown to decrease hepatic insulin resistance. Lipid-laden hepatocytes can store nonpolar hepatotoxic agents. The ensuing susceptibility to oxidant damage leads to lipid peroxides and unsaturated keto-aldehydes. The latter can cause hepatic necrosis and enhanced fibrogenesis. Starting from a simple steatosis NAFLD, can proceed to steatohepatitis and fibrosis. Cirrhosis and/or hepatic carcinoma can be terminal complications.[8]

In India, the approximately 32 million diabetics in the year 2000 is predicted to jump to 69.9 million by 2025. In these circumstances, NAFLD will assume alarming proportions.[4] Metabolic syndrome has NAFLD as a component in addition to visceral obesity, hypertension, dysglycemia, and dyslipidemia.[5–7] Underlying insulin resistance (IR), oxidative stress, and proinflammatory cytokines are implicated as causes of both steatohepatosis and steatohepatitis.[5,9,10] It is now recognized that IR is present even in NAFLD patients with euglycemia who are of normal weight.

The spectrum of NAFL and its complications being wide, management of the disease is challenging. Weight reduction, ursodeoxycholic acid, clofibrate, gemfibrozil, vitamin E, metformin, and betaine have been recommended. But none of these are conclusively effective, and well-controlled clinical trials are called for.[11] The effect on hepatic morphology of treatment of obesity with fasting, reducing diets, and small bowel bypass has been studied.[12]

In India, hepatoprotective medicinal plants and their formulations have been traditionally used in Ayurveda for the prevention and treatment of diverse liver diseases. *Picrorhiza kurroa* has been commonly used and well investigated for the treatment of jaundice.[13] The plant has also been shown to be hepatoprotective in various animal models of hepatotoxicity like carbon tetrachloride, d-galactosamine, paracetamol, and thioacetamide.[14–17] It plant has been shown to be hydrocholerectic in a biliary fistula model in dogs and humans.[18] In a double-blind trial in patients with viral hepatitis, *P. kurroa* rhizome powder was shown to be hepatoprotective by Vaidya *et al*.[19] Earlier, a commonly used Ayurvedic formulation – Arogyavardhini – containing 50% *P. kurroa* was also found effective in a double-blind trial in viral hepatitis.[20] It was therfore considered desirable to study *P. kurroa* in an animal model of NAFLD. *Silybum marianum* is the most well-researched plant in the treatment of liver diseases. Silymarin has been shown to have significant anti-inflammatory effects on hepatic tissue. Several studies have demonstrated a variety of anti-inflammatory effects, including mast cell stabilization, inhibition of neutrophil migration, and Kupffer cell inhibition.[21–25] It was used as a control in the present study. Significant effort was put into creating an animal model of NAFLD.

## MATERIALS AND METHODS

### Chemicals

**Figure 1:**
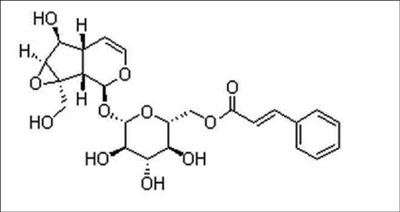
Picroside I

Picroside I [Figure 1] was kindly supplied by the Central Drug Research Institute (CDRI), Lucknow. Silymarin was purchased from Sigma Chemicals Co. USA. All other chemicals used were of analytical grade. Biochemical kits for cholesterol (CHO), triglyceride (TG), high-density lipoprotein (HDL), alanine aminotransferase (ALT), alkaline phosphatase (ALP) for lipid assays, and liver tests used for the study were purchased locally from Raichem, Merck and Ranbaxy, India. Erba Chempro Biochem Analyzer was used for the estimation of all biochemical parameters.

### Plant material

The medicinal plant *P. kurroa* was identified and its rhizomes collected from the Himalayan region. It was authenticated and standardized at the Zandu Pharmaceutical Works Pvt. Ltd., Mumbai, who have been using the plant for many decades. The pharmacognosy, markers, and other characteristics were as per the WHO guidelines. The voucher specimen was deposited in their herbarium.

### Preparation of plant extract

After proper cleaning, the *P. kurroa* rhizomes were subjected to soxhlet extraction by the ethyl alcohol–water (50:50) method. Each extraction was done thrice for 4 hours each. The total filtrate of the extract was put up for distillation. The filtrate was finally spray-dried to yield a powder form. The yield of the extract was 33.95% w/w. This hydroalcoholic extract (HAE) of *P. kurroa* was then stored in a vacuum dessicator. During the experiment, an appropriate aliquot of the crude extract was diluted with carboxy methyl cellulose (CMC) before administration to the animals.

### Experimental animals

Male Wistar rats, inbred at M/s. Bharat Serum and Vaccine Pvt. Ltd., Mumbai, India, were procured after obtaining clearance for the experiment from the Institutional Animal Ethics Committee of SNDT University. Animals weighing 200–250 g were reared on a balanced laboratory diet and given clean water *ad libitum*. They were kept at 20 ± 2°C, 65–70% humidity, and day/night cycle (12 h/12 h). “Amul” butter fresh batch available in the market was the HFD used for the study.

### Experimental design

#### Methods for establishing NAFLD model and assessing the effect of HAE P. kurroa

The animals were fasted overnight for 16 hours with free access to water. At the end of the fasting period, blood was collected from the retro-orbital plexus under mild anesthesia to evaluate basal values for the biochemical estimations – TG, CHO, HDL, ALT and ALP. The animals were then randomized into five groups (*n* = 6/group).

**Group 1:** The animals were fed *ad libitum* for 2 weeks. After 2 weeks, 0.5ml CMC, the vehicle was given orally along the same diet for 4 weeks.

**Group 2:** The animals were fed on 30% HFD orally for 6 weeks.

**Group 3–5:** The animals were fed on 30% HFD for 2 weeks. After 2 weeks, along with 30% HFD, Silymarin 50 mg/kg (Group 3), HAE *P. kurroa* 200 mg/kg (Group 4), or HAE *P. kurroa* 400mg/kg (Group 5) were given p.o. for 4 weeks.

Body weight of the animals was taken on a weekly basis. Retro-orbital bleeding was again conducted at the end of 2nd and 6th week after overnight fasting for the biochemical tests. At the end of the treatment period, the animals were sacrificed by cervical decapitation, and the liver assessed for histopathology and lipid content.

#### Assessment of the biochemical and histopathological criteria

The liver was dissected and immediately rinsed in ice cold saline. The liver was weighed and gross examination was carried out. It was then stored in 10% formalin and later processed for histopathological studies by hematoxylin and eosin (H and E) staining.

Damage produced in the liver structure in the form of degeneration, necrosis, and fibrosis was graded as follows:

#### Degeneration

Gr 0, no degeneration; Gr 1, few fat cells per field; Gr 2, more than 100 fat cells per field;

Gr 3, one or two rows of fat cells per lesion; Gr 4, extensive centrilobular and midzonal fatty change with ballooning degeneration.

#### Necrosis

Gr 0, no necrosis; Gr 1, centrilobular necrosis or 1 or 2 cells per lesion; Gr 2, centro-centri bridging necrosis; Gr 3, massive centrilobular necrosis; Gr 4, massive centrilobular necrosis with necrotic tissue bridging the central veins.

#### Fibrosis

Gr 0, no fibrosis; Gr 1, central necrosis, mild periportal fibrosis; Gr 2, presence of fibrous tissue in portal tract area only, along with other change mentioned for Grade 1. Gr 3, fibrous tissue insinuating surrounding hepatic parenchyma; Gr 4, formation of pseudolobule by this insinuated fibrous tissue.

Lipid content of the liver was assessed by using Folch *et al*. method.[26] Liver function tests - TG, CHO, HDL, ALT, ALP were conducted spectrophotometrically using standard kits.

### Statistical analysis

Statistical analysis was carried out using one way analysis of variance (ANOVA) test to assess the statistical significance between control, HFD and intervention groups. The difference between the mean ± standard error of the biochemical values was tested for significance.

## RESULTS

### Phytochemical analysis of the raw plant material and crude extract of HAE *P. kurroa*

The total ash content of raw material of *P. kurroa* was estimated to be 4.53%. Bitter principles (picrosides) were dominant in the crude *P. kurroa* material (20%), while HAE *P. kurroa* showed 6.54% bitters.[27]

Microbial analysis by colony forming units (CFU/10 g) was carried out for *Escherichia coli*, Staphylococcus, Streptococcus, Salmonella, Moulds, and Yeast. The extract used for intervention was free of microbes. Standardization of the selected *P. kurroa* for quality and purity was confirmed prior to the study as per WHO criteria.

### Effect of the HAE *P. kurroa* on the rat model of NAFLD with high fat (30%) diet

**Figure 2:**
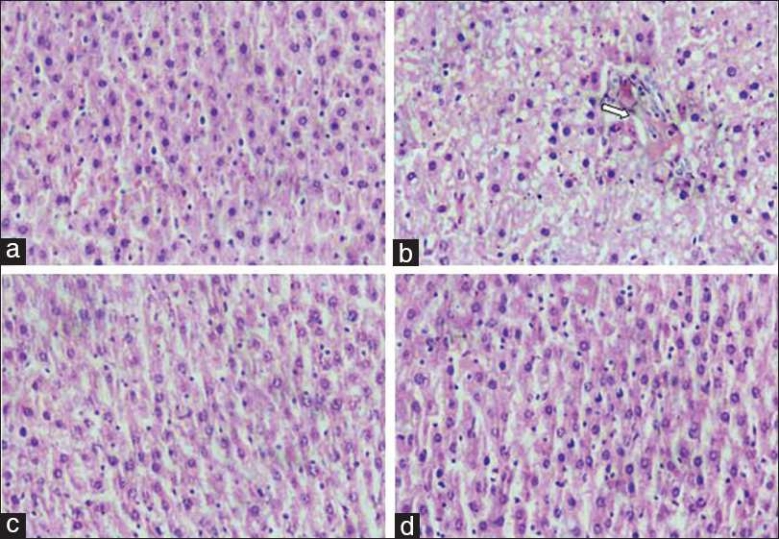
(a) Normal architecture of rat hepatic lobule (×40) H & E stained. (b) Fatty liver tissue with marked diffuse granular degeneration and mild multifocal biliary hyperplasia (×40). (c) Histology of liver tissue treated with standard silymarin showing normal architecture (×40) H & E stained. (d) Histology of liver tissue treated with P. kurroa 400 mg/kg showing normal architecture (×40) H & E stained.

Gross examination of the liver of the vehicle control group showed normal reddish colour compared to the pale colour in the HFD group. Histopathology of the liver showed normal architecture in the vehicle control group, while fatty infiltration with granular degeneration and mild multifocal biliary hyperplasia was observed in the diet control group. It is of interest to note that the group treated with *P. kurroa*, like the silymarin group, showed minimal hepatic damage and a distinct preservation of structure and architecture of liver tissue [Figures 2a–d].

**Figure 3:**
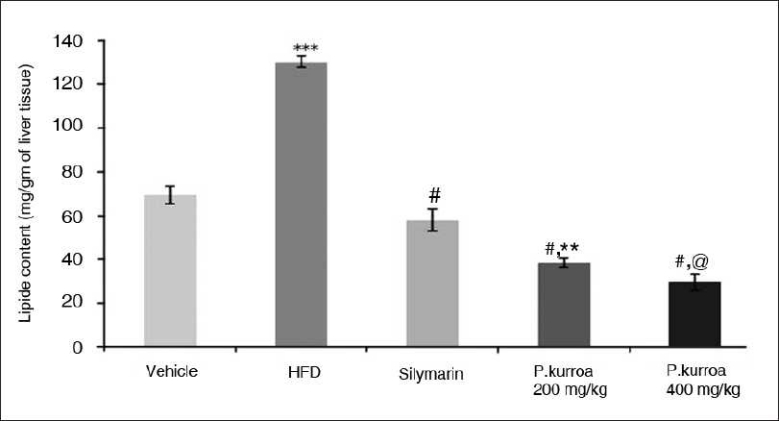
Lipid content in mg/gm of liver tissue. Vehicle vs HFD *** *P*<0.001; HFD vs Silymarin, P.kurroa 200 mg/kg and P.kurroa 400 mg/kg # *P*<0.001; Silymarin vs P.kurroa 200 mg/kg ** *P*<0.01; Silymarin vs P. kurroa 400 mg/kg @ *P*<0.001.

Neither body weight nor the weight of the liver showed significant difference between groups during the course of the study. Despite lack of change in liver weights, there was a marked rise in lipid content of liver tissue [Figure 3] in the HFD model group (130.07± 2.6 mg/g) compared to the standard silymarin group (57.71 ± 5.08mg/g). The vehicle control without any HFD had lipid content of 69.2 ± 3.98mg/g. Both the *P.kurroa*-treated groups showed a decrease in lipid content in their liver tissue. The group treated with 400mg/kg *P. kurroa* (29.44 ± 3.47 mg/g) showed greather reduction in lipid content than the silymarin group (*P*<0.001) though the change was not significant compared to the 200 mg/kg *P. kurroa* group.

**Figure 4:**
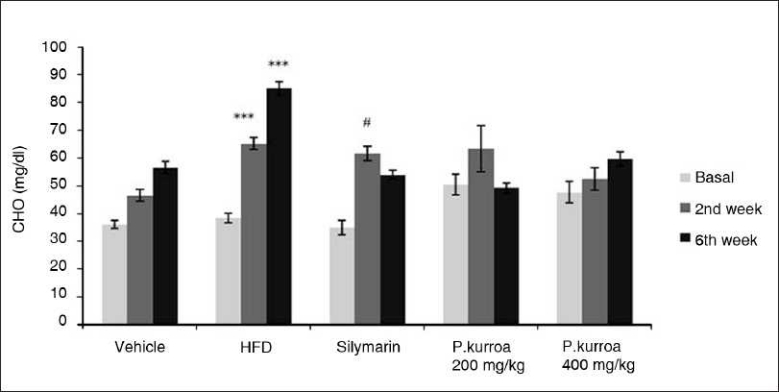
Comparison of serum cholesterol levels. HFD basal vs HFD 2^nd^ and HFD 6^th^ week values *** *P*<0.001; Silymarin basal vs Silymarin 2^nd^ week values # *P*<0.001.

Serum cholesterol levels in all groups are shown in Figure 4. There was an age-related progressive increase in serum cholesterol in the vehicle control group as shown in Figure 3. In the HFD group, the age-related increase in serum cholesterol was further amplified. Compared to base values, there was a significant increase in the cholesterol values at the 2nd and 6th weeks (38.3 ± 1.75, 65.13 ± 2.04, 84.97 ± 2.4mg/dl, respectively). In the silymarin group, there was a significant increase in cholestrol levels at the 2nd week (61.45 ± 2.62mg/dl) compared to the base value (34.82 ± 2.61mg/dl). However, by the 6th week the silymarin group’s age-related rise in serum cholesterol levels had been attenuated (53.9 ± 1.62mg/dl) compared to those in the HFD group. Similarly the values of the group treated with HAE *P. kurroa* 200 mg/kg were further reduced by the end of the 6th week (49.08 ± 1.87mg/dl) compared to their 2nd week value (63.21 ± 8.39mg/dl). However, in the group treated with HAE *P. kurroa* 400mg/kg, attenuation in the value of serum cholesterol was not observed (59.52 ± 2.7 mg/dl).

**Figure 5:**
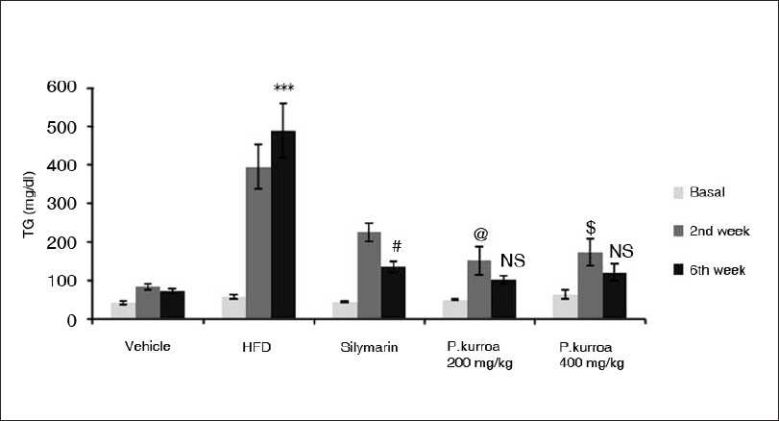
Comparison of serum triglyceride levels. HFD basal *vs* HFD 6^th^ week values ***P<0.001; Silymarin 2^nd^ week vs Silymarin 6^th^ week values # *P*<0.01; *P.kurroa* 200 mg/kg basal *vs* *P.kurroa* 2^nd^ week values @ *P*<0.05; *P.kurroa* 200 mg/kg 2^nd^ week *vs* 6^th^ week values - No significance (NS); *P.kurroa* 400 mg/kg basal *vs* *P.kurroa* 2^nd^ week values $ *P*<0.05; Pk 400 mg/kg 2^nd^ week *vs* *P.kurroa* 6^th^ week values – NS.

Serum triglyceride levels are given for all groups in Figure 5. An age-related increase in serum TG was seen in the vehicle control group from basal to the 2nd and 6th weeks (41.28 ± 5.03, 82.07 ± 7.24, 71.1 ± 5.62 mg/dl). In the HFD group, there was a significant amplification in the age-related increase in levels of TG in the 2^nd^ week – 393.99 ± 56.6 mg/dl and in the 6th week – 487.75 ± 70.94mg/dl compared to the basal value – 56.55 ± 4.3mg/dl. However, in the silymarin group, the 6th week value showed attenuation in the age-related rise in TG levels (basal – 42.46 ± 2.63mg/dl, 2nd week – 223.82 ± 22.63mg/dl, 6th week – 133.52 ± 14.7mg/dl). In the *P. kurroa*-treated groups, the values of the 6th week analysis showed a decrease compared to the 2nd week value though the effect was not significant.

**Figure 6:**
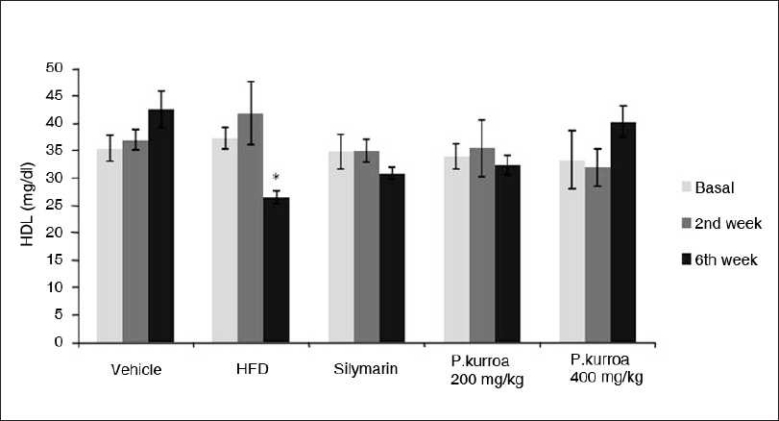
Comparison of serum high density lipoprotein levels. HFD 2^nd^ week *vs* HFD 6^th^ week values * *P* < 0.05.

The HDL levels are shown for the groups in Figure 6. In the vehicle control group, the values of HDL in the 2nd and 6th week of the analysis were 37 ± 1.86, 42.5 ± 3.38mg/dl, respectively. However, a marked decrease in the HDL levels was observed in the HFD group by the 6th week (26.5 ± 1.1 mg/dl). In the silymarin group and in the *P. kurroa* 200mg/kg, there was no remarkable change in the HDL levels by the 6th week of the intervention. However, it was of interest to observe that the HDL levels in the HAE *P. kurroa* 400mg/kg did not show a decline in the 6th week, but was comparable to that seen in the vehicle control group.

**Figure 7:**
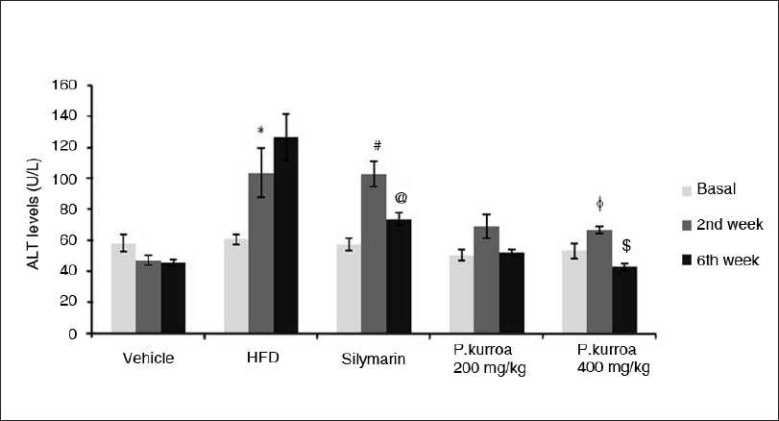
Comparison of serum Alanine Amino Transferase levels. HFD basal *vs* HFD 6^th^ week values *P<0.05; Silymarin basal *vs* Silymarin 2^nd^ week values # *P* < 0.001; Silymarin 2^nd^ week *vs* Silymarin 6^th^ week values @ *P* < 0.001; *P.kurroa* 400 mg/kg basal *vs* *P.kurroa* 2^nd^ week values Φ *P*<0.05; *P.kurroa* 400 mg/kg 2^nd^ week *vs* *P.kurroa* 6^th^ week values $ *P*<0.001.

After the two week induction period, a marked rise in ALT levels was observed in the HFD group and also in the group treated with silymarin. In the HFD model and silymarin group, there was a significant increase in ALT levels at the 2nd week of the study (103.43 ± 16.04 U/L; 102.65 ± 19.45 U/L) compared to the vehicle control (46.95 ± 3.14 U/L). At 6 weeks, the HFD group showed a further increase in ALT values (126.65 ± 14.98 U/L), whereas in the silymarin group the level was reduced (73.65 ± 3.89 U/L). HAE *P. kurroa* 200 and 400 mg/kg showed dose-dependent results (51.81 ± 2.13 and 42.86 ± 2.35 U/L, respectively) [Figure 7].

**Figure 8:**
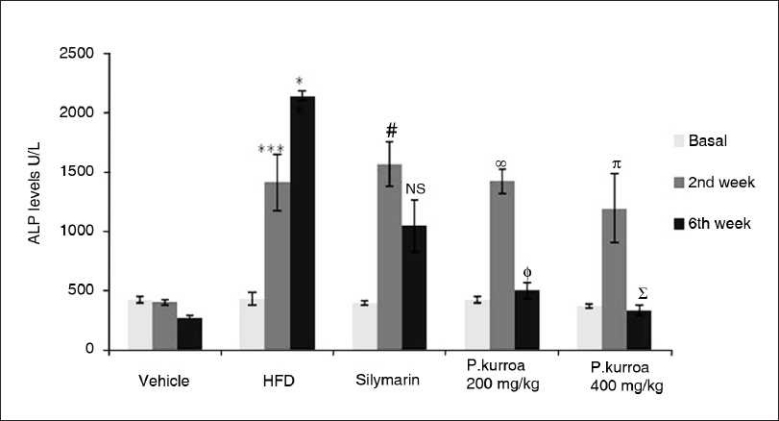
Comparison of serum Alkaline Phosphatase levels. HFD basal *vs* HFD 2^nd^ week ***P<0.001; HFD 2^nd^ week *vs* HFD 6^th^ week values *P<0.05; Silymarin basal *vs* Silymarin 2^nd^ week values # *P*< 0.001; Silymarin 2^nd^ week *vs* Silymarin 6^th^ week values - NS; *P.kurroa* 200 mg/kg basal *vs* *P.kurroa* 2^nd^ week values @ *P*<0.001; *P.kurroa* 200 mg/kg 2^nd^ week *vs* *P.kurroa* 6^th^ week values Φ *P*<0.001; *P.kurroa* 400 mg/kg basal *vs* *P.kurroa* 2^nd^ week values π *P*<0.05; *P.kurroa* 400 mg/kg 2^nd^ week *vs* *P.kurroa* 6^th^ week values Σ *P*<0.01.

The ALP levels in HFD, silymarin, *P. kurroa* 200 and 400 mg/kg groups were significantly higher in the 2nd week (1408.08 ± 233.74, 1563.5 ± 189.23 U/L, 1417.10 ± 253.58, 1192.05 ± 718.18 U/L, respectively) compared to the vehicle control group (402.23 ± 22.01 U/L; *P*<0.001). At the end of the study, the ALP values showed a marked rise in the HFD group compared to the 2nd week (2136.17 ± 41.01 U/L). Although a reduction in ALP levels was observed by the 6th week of treatment with silymarin, the values had not normalized. However, in both the *P. kurroa*-treated groups ALP levels not only reduced but had returned to normal values [Figure 8].

## DISCUSSION

NAFLD is a component of metabolic syndrome which comprises central obesity, hypertension, impaired fasting glucose (IFG), impaired glucose tolerance (IGT), hypertriglyceridemia, and low HDL.[28] Metabolic syndrome is a risk factor for type II diabetes mellitus, coronary artery disease, and cerebro-vascular disease.[29] NAFLD has also been implicated in the occurrence of insulin resistance and diabetes mellitus.[30–32] The latter conditions have reached globally epidemic proportions. India has the world’s highest incidence of diabetes mellitus. However, insufficient epidemiological data exist on the prevalence of NAFLD in the subcontinent. But clinical impressions and some studies suggest it has a high prevalence.[33,34] The spectrum of NAFLD is wide, ranging from simple and reversible fat accumulation in hepatocytes, eventually leading to irreversible fibrosis, and even terminal cirrhosis or cancer. There are thus, challenges and opportunities for the management and prevention of the disease. Although weight reduction, ursodeoxycholic acid, clofibrate, gemfibrozil, vitamin E, metformin, and betaine have been recommended as modalities of therapy, none of them are very effective.[35–40] Ancient Ayurvedic literature and current basic and clinical research have identified several Indian medicinal plants / formulations with hepatoprotective effects.[14,20,41] The purpose of the present study was to investigate the hepatoprotective potential of a standardized extract of *P. kurroa* in an HFD-induced animal model of NAFLD to demonstrate a reversal of hepatic lesions, lipid, and enzyme changes. Silymarin, a flavolignan extracted from the seeds and fruit of *Silybum marianum*, was used as a positive control.

Extracts of roots and rhizomes of *P. kurroa* have shown hepatoprotective activity in diverse models of liver toxicity.[42–44] *P. kurroa* contains iridoid glycosides (including picroside I, II, III, pikuroside, kutkoside and 6-feruloyl catalpol), cucurbitacin glycosides, androsin, apocynin, and other organic acids such as vanillic and cinnamic acids. It is pertinent to note its ayurvedic properties of tikta rasa, laguruksha guna, and katuvipaka. Based on these properties, one may anticipate its pharmacodynamic activity on lipids specifically related to lipid disorders.[45] Picroside I has earlier been shown to be active in several models of liver toxicity.[43]

In the present study markers and pharmacologically active compounds of the selected medicinal plants were demonstrated by chromatographic methods. The finger print pattern of the HAE *P. kurroa* was carried out by TLC, HPTLC, and HPLC for the standard compound, Picroside I, at CDRI, Lucknow. The chromatographic identity of the experimental extracts with pure standards validated the extraction methods, and gave relevance to the interventional studies, with the putative actives in the extracts. Picroside I was detected by HPLC in the standardized extracts (data not shown).

Development of an animal model of NAFLD was a formidable challenge. For interventional studies with the selected medicinal plant, the model had to be simple, suitable, and economical. Studies have demonstrated that monounsaturated and saturated fatty acids (MUFA and SFA) favor adiposity by increasing lipogenesis.[46] Hence, the commonly used form of animal fat – butter – which is rich in saturated fatty acids was selected. An HFD comprising various concentrations of butter was used to induce NAFLD in the rat model and 30% of high fat was found suitable for the study. The current animal model has displayed a close resemblance to the clinical entity of NAFLD in terms of histopathological and biochemical changes. Earlier attempts to develop NAFLD animal models with other hepatotoxic agents met with less success (unpublished data).

In the present study, we have shown that 30% HFD significantly increased the lipid content of the liver tissue without affecting the body weight or liver weight. Raised serum lipid levels were also observed. A rise in serum ALT and ALP – an important feature of NAFLD – was observed in this model. Pan *et al*, have demonstrated that HFD can induce oxidative stress with extensive liver steatosis in rats.[47]

Histological findings in NAFLD range from steatosis, hepatocyte ballooning, mild-lobular inflammation, and perisinusoidal collagen deposition.[48] In the rat HFD model (30%) used in the present study, the histopathology of the liver sections showed fatty changes. As this model generated hepatic lesions and blood chemistry changes similar to those in NAFLD it was adopted to study interventional effects of extracts of the selected medicinal plant.

Changes in body weight in all groups showed age-related increases, but differences were not significant between groups. Despite decreases in weight gain, there was no statistical significance in body weight values obtained with plants compared to the vehicle control or the diet model. Liver weight expressed as g/100g of body weight did not show any significant difference between the control, diet model, and plant intervention groups.

A twofold increase in the liver-lipid content was observed with HFD justifying the suitability of the model. The group on silymarin showed a significant reduction in the lipid content of the liver compared to the diet model group. The *P. kurroa* intervention group showed a reduction in lipid content with dose response directionality.

The histopathological studies corroborated the above statements of reduction in lipid content by a parallel and significant morphological normalization of liver architecture and a marked reduction in fatty infiltration in hepatocytes. Both *P. kurroa* and its active principles have been shown to have hydrocholeretic activity and to increase bile production.[18] Currently therapy-induced reduction in serum cholesterol and fatty liver are mainly ascribed to bile salt sequestrants such as the resin – cholestyramine. Resin bound bile salts do not enter the enterohepatic circulation, and this assists further biliary excretion of bile salts, the breakdown products of cholesterol. The mechanism of lipid-lowering effects of the active principles of *P. kurroa* needs to be explored. The plant’s anti-inflammatory effects may be an additional benefit when steatosis evolves into steatohepatitis.[49]

Both silymarin and HAE *P. kurroa* showed a significant hypocholesterolemic effect compared to the diet model. The distinct rise of cholesterol in the second week was not reflected in the HDL change. This finding suggests that the specific lipoprotein under the influence of 30% HFD should be characterized. By the 6th week, the HFD model showed a significant (*P*<0.001) reduction in HDL as compared to vehicle control. However it was interesting to note that *P. kurroa* at 400 mg/kg showed an HDL value of 40.27 ± 2.85mg/dl, a significant rise compared to the value for the HFD group (26.50 ± 1.1mg/dl). The value with *P. kurroa* almost approached the 6th week value of the vehicle control. These findings need to be pursued in-depth with other models of hyperlipidemia and atherosclerosis as elevation of HDL levels is considered quite difficult to achieve. Low HDL is an important risk factor for coronary heart disease.[50]

Both intervention doses and silymarin reduced TG levels significantly compared to the HFD group. The elevation of transaminase reflects necrosis of hepatocytes.[51] ALT in particular is a more specific index of hepatic necrosis. With silymarin, ALT levels were reduced significantly compared to HFD. However, the effect observed with HAE *P. kurroa* was more effective, bringing down ALT levels to values comparable to vehicle control at the higher 400mg/kg dose. This finding needs to be pursued further in *in vitro* hepatocyte cultures, and other models of mild to moderate hepatic injury. The anti-inflammatory activity of this plant should also be evaluated *vis-à-vis* the degree of necrosis-induced inflammation *in vivo*. In particular, first-phase proteins and release of proinflammatory cytokines, which occur as a reaction to cell death, should be studied.[52]

ALP reflects cholestatic damage that may be induced by hepatotoxic agents.[53,54] Although intrahepatic biliary stasis was observed in the liver biopsies of HFD group, it was attenuated in the treated groups. This clearing of cholestasis along with low ALP levels in the *P. kurroa*-treated groups confirms the plant’s hepatoprotective activity. In both the *P. kurroa* dose groups, 6th week serum ALP levels were significantly lower compared to those observed in the silymarin group. *P. kurroa* showed highly significant reduction in ALP at both dose levels. The values almost approached basal values of ALP and showed dose–response relationship. Further studies with cholestatic models should be carried out.

## CONCLUSIONS

The present study has evolved an appropriate animal model with several features of NAFLD, correlating with those seen in humans. Intervention with standardized plant extracts of *P. kurroa* regressed several features of NAFLD like lipid content of the liver tissue, morphological regression of fatty infiltration, hypolipidemic activity, and reduction of cholestatis. The present study should be pursued further for drug development based on reverse pharmacology for management of NAFLD.
